# Effectively Quantifying the Performance of Lower-Limb Exoskeletons Over a Range of Walking Conditions

**DOI:** 10.3389/frobt.2018.00061

**Published:** 2018-06-27

**Authors:** Daniel F. N. Gordon, Graham Henderson, Sethu Vijayakumar

**Affiliations:** Institute of Perception, Action, and Behaviour, School of Informatics, University of Edinburgh, Edinburgh, United Kingdom

**Keywords:** *exoskeletons*, gait metrics, stability, metabolic energy, control, musculoskeletal modelling

## Abstract

Exoskeletons and other wearable robotic devices have a wide range of potential applications, including assisting patients with walking pathologies, acting as tools for rehabilitation, and enhancing the capabilities of healthy humans. However, applying these devices effectively in a real-world setting can be challenging, as the optimal design features and control commands for an exoskeleton are highly dependent on the current user, task and environment. Consequently, robust metrics and methods for quantifying exoskeleton performance are required. This work presents an analysis of walking data collected for healthy subjects walking with an active pelvis exoskeleton over three assistance scenarios and five walking contexts. Spatial and temporal, kinematic, kinetic and other novel dynamic gait metrics were compared to identify which metrics exhibit desirable invariance properties, and so are good candidates for use as a stability metric over varying walking conditions. Additionally, using a model-based approach, the average metabolic power consumption was calculated for a subset of muscles crossing the hip, knee and ankle joints, and used to analyse how the energy-reducing properties of an exoskeleton are affected by changes in walking context. The results demonstrated that medio-lateral centre of pressure displacement and medio-lateral margin of stability exhibit strong invariance to changes in walking conditions. This suggests that these dynamic gait metrics are optimised in human gait and are potentially suitable metrics for optimising in an exoskeleton control paradigm. The effectiveness of the exoskeleton at reducing human energy expenditure was observed to increase when walking on an incline, where muscles aiding in hip flexion were assisted, but decrease when walking at a slow speed. These results underline the need for adaptive control algorithms for exoskeletons if they are to be used in varied environments.

## 1. Introduction

Increasingly, exoskeletons are being used to great effect for the rehabilitation of people with lower-limb pathologies (Dollar and Herr, 2008). Additionally, exoskeletons are being developed as assistive tools to reduce the metabolic cost of walking, with some recent advances in state-of-the-art soft exosuits (Panizzolo et al., 2016) demonstrating energy savings of more than 20% (Quinlivan et al., 2017). However, exoskeletons used for these purposes are largely restricted to supervised clinical or research settings, where time and care is taken to ensure that the behaviour of the exoskeleton and the nature of the rehabilitation or assistive regime is well-suited to the subject in question (Wolff et al., 2014). The use of exoskeletons in a real-world setting, e.g. to assist the elderly in everyday life, is made difficult by the number of variables to consider as a human walks in an uncontrolled environment — for example, walking speed, or whether the subject is walking on an incline. Each of these variables can affect the gait pattern of an individual, and therefore the optimal torques to be applied by an assistive device. If exoskeletons are to become widely used devices outside of a clinical setting it is important that a suitable control paradigm is developed that, either implicitly or explicitly, applies assistance that accounts for these variables.

Current control paradigms frequently use normalised kinematic trajectories (Riener et al., 2010), muscle amplification (Ferris and Lewis, 2009), or finite state controllers (Blaya and Herr, 2004). The respective issues with these paradigms are that the kinematic trajectory might not be appropriate for the user’s task or their environment, the muscle firing patterns may be abnormal, and there are a large number of parameters to tune.

It is known that the human neuromuscular system optimises stability (Kuo and Donelan, 2010). By studying the effect of different walking contexts and constant perturbations (applied via an exoskeleton) on healthy walking, it is posited that there will be an underlying invariant metric that reflects the optimisation of the stability of human gait. Once identified, this metric can then be optimised as part of an exoskeleton control paradigm which provides assistance while maintaining balance, implicitly accounting for the effects of changing walking context and varying exoskeleton assistance. Previous work has been carried out to determine what effect walking speed (Winter, 1984; Stansfield et al., 2001; Orendurff et al., 2004), the environment (Lay et al., 2006; Franz and Kram, 2012), and exoskeleton forces have (Lewis and Ferris, 2011; Lenzi et al., 2012; Martelli et al., 2014) on a user’s gait but these are constrained by using limited metrics and, for the work done on exoskeletons, limited walking contexts.

The human neuromuscular system also optimises energy efficiency (Kuo and Donelan, 2010). In a similar analysis to what is outlined above, the effect of different walking contexts and exoskeleton forces on healthy walking can be measured in terms of the metabolic energy consumed by the muscles of the subject. This relationship could be optimised as part of a model-based exoskeleton control paradigm, alongside a stability metric, where the aim is to reduce total human energy expenditure or, alternatively, target specific groups of muscles for rehabilitation or assistance. Once known, this relationship can be used to inform how exoskeleton controllers are implemented for use in real-world settings where steady, flat walking is not guaranteed.

In this study, a neuromuscular human and exoskeleton model is presented. Experimental data was collected using a unique setup, combining kinematic, kinetic, and exoskeleton angular and torque data. Using this data, stability metrics and metabolic energy consumption were compared between three walking scenarios: walking without an exoskeleton, walking with an exoskeleton in transparent mode, and walking with an exoskeleton in assistive mode. For each of these scenarios five different walking contexts were investigated: walking at baseline speed, walking up an incline, walking down an incline, fast walking, and slow walking. To carry out the analysis a range of spatial and temporal, kinematic, kinetic, and dynamic gait metrics (such as centre of mass displacement) were selected.. The selected metrics were compared to identify those which demonstrated the most invariance and therefore would be suitable for optimising in an exoskeleton control paradigm. In addition, metabolic energy consumption was calculated and is reported for a subset of muscles crossing the hip, knee and ankle joints, and the effect of variations in walking context and exoskeleton assistance level on these representative muscles is discussed.

## 2. Material and Methods

### 2.1. Model Development

**Figure 1:**
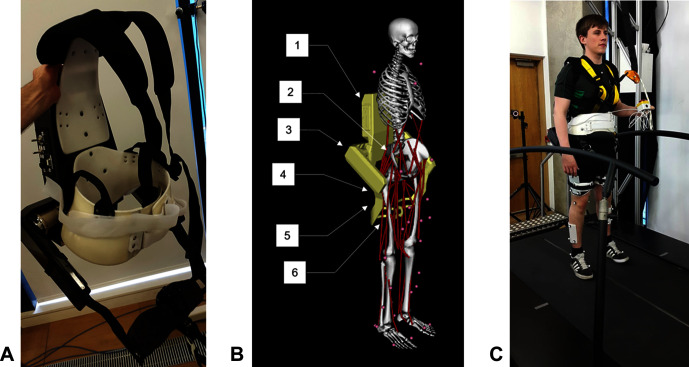
**(A)** The Active Pelvis Orthosis (APO). **(B)** An overview of the APO OpenSim model’s constraints, bodies, and degrees of freedom: (1) APO backpack, (2) Weld constraint between APO backpack and pelvis, (3) right APO group body (houses the actuators), (4) APO free joint (6 DOF), (5) right APO link, (6) weld constraint between APO link and femur. **(C)** The experimental setup. This image is published with the written informed consent of the depicted individual. ©IEEE 2017

The exoskeleton which we use to provide assistance is the Active Pelvis Orthosis (APO), a revised version of the device presented by Giovacchini et al. (Giovacchini et al., 2015) (see Figure 1A). This exoskeleton was developed at The BioRobotics Institute of Scuola Superiore Sant'Anna (Pisa, Italy); the technology is currently licensed to IUVO Srl (http://www.iuvo.company, Pontdera, Italy). The APO provides a force applied to the thighs of the user transmitted via two carbon fibre lateral arms which are actuated by series elastic actuation units.

The APO developers adapted work by Ronsse et al. (Ronsse et al., 2011) to construct a high-level assistive controller, which generates a zero-delay estimate of the hip angles during gait and calculates a desired torque which is proportional to the estimated change in hip angle. A constant virtual stiffness parameter is used to calculate the torque necessary to drive the user’s joint positions towards their expected future values. The APO can also be operated in “transparent mode”, where the system provides no assistance to the user and the joints are free of resistance.

We developed a unique model of a human subject wearing the APO (see Figure 1B) using the software OpenSim (Delp et al., 2007). We took a pre-existing OpenSim human model with 92 muscles and 23 degrees of freedom, known as the gait2392 model (Yamaguchi and Zajac, 1989; Delp et al., 1990a; Anderson and Pandy, 1999a; Anderson and Pandy, 2001), and constrained the APO to it with three weld constraints, located between the backpack and the pelvis and between the two links and the femur. The APO mass and inertia properties were imported from a CAD model provided by IUVO.

### 2.2. Experimental Protocol

Data was collected for each subject while they walked on a treadmill in a variety of walking contexts and exoskeleton assistance scenarios. Reflective markers were attached to each subject to accurately track their movements using a six camera motion capture system (Vicon, Oxford, UK). The marker set used was adapted from the Cleveland marker set and consisted of 33 markers, 8 of which were solely used for the purpose of scaling the dynamic model. Ground reaction forces and moments were collected using a six axis, split belt instrumented treadmill (Motekforce Link, Amsterdam, Netherlands). The torques applied by the APO were measured directly from the device. Figure 1C demonstrates the experimental set up.

To capture data in different walking contexts, a script was implemented in the Motek D-Flow software to programmatically change the speed or incline of the treadmill appropriately. Each subject was made to walk in five different walking contexts, as follows: at baseline walking speed with no incline (BW), at baseline walking speed with an incline of 5 degrees (UW), at baseline walking speed with an incline of −5 degrees (DW), at a fast walking speed with no incline (FW), and at a slow walking speed with no incline (SW). The baseline walking speed used for the BW context was calculated using the principle of dynamic similarity as described by the Froude number (Vaughan and O'Malley, 2005):

(1) $$v=\sqrt{F_{r}\cdot g\cdot L},$$

where v is the baseline speed, $F_{r}$ is the Froude number (chosen to be 0.1), g is gravitational acceleration ($9.81\text{ m}/{\text{s}}^{2}$), and L is leg length (as measured from the greater trochanter to the medial malleolus). The speeds for FW and SW were calculated by adding and subtracting 20% to the baseline speed respectively. Each context was timed to last 135 s, with data collection triggered to happen after 120 s to allow for the participant to become accustomed to the context. For time synchronisation, the D-flow script sent a command to a relay box which simultaneously triggered the Vicon system, Motek treadmill and APO to begin recording data while accounting for internal delays. The kinematics, ground reaction forces and moments, and APO data were captured at 100 Hz, 600 Hz, and 100 Hz respectively.

The contexts were repeated for 3 different assistance scenarios: one without wearing the APO (NE), one wearing the APO set in transparent mode (ET) and one wearing the APO set in assistive mode (EA) with the virtual stiffness set to 15 Nm/rad. For each subject, a static pose was collected in both the NE and ET assistance scenarios.

### 2.3. Post-Processing

Before the data could be analysed, several post-processing steps had to be undertaken. Any gaps in the raw recorded motion capture data were filled using Vicon’s software Nexus. A combination of the built in algorithms were used including the spline fill, the pattern fill, and the cyclic fill. The MoNMS toolbox (Mantoan et al., 2015) was used for the remainder of the motion capture data processing. The marker trajectories were first low-pass filtered with a zero-lag 4th order Butterworth filter and then transformed from the Vicon axis system into the OpenSim axis system.

For the ground reaction forces and moments, custom scripts were written in MATLAB. The first step was to compensate for data collected when the treadmill was tilted, and therefore causing gravity to work in a different direction to the force plate sensors. The ground reaction forces were then filtered using a zero-lag 4th order Butterworth filter with a 6 Hz cut-off. For the next step a threshold filter was applied to the ground reaction forces and moments that set all values equal to zero when the vertical force was less than 40 N. This was implemented because the CoP values were noisy when the vertical ground reaction forces were low. Additionally, it filtered out any noise in the force measurements during the swing phase of the gait cycle when there should be no forces applied to the foot. After applying the threshold, the CoPs were calculated and the global force plate moments were converted into free moments around the foot. Finally, the D-flow axis system was transformed to the OpenSim axis system.

### 2.4. APO Joint Misalignment

The problem of joint misalignment is well known when dealing with physically coupled systems, e.g. humans wearing exoskeletons (Malosio et al., 2011; Jarrassé and Morel, 2012; Zanotto et al., 2015). If joints are perfectly aligned, exoskeleton forces can be modelled as equal and opposite torques applied to the relevant exoskeleton and human bodies. However, the presence of joint misalignment results in imperfect transmission of torque from the exoskeleton to the human user (Schiele and van der Helm, 2006), ultimately resulting in reduced torque about the human joint and the introduction of undesirable forces parallel to the human limb, which can cause discomfort or unintended changes to muscle activation patterns (Hidler and Wall, 2005). The design of the APO is such that the exoskeleton joints should closely align with those of the human (Giovacchini et al., 2015), however it is not realistic to expect perfect alignment. By fixing a reflective marker on the rear of the exoskeleton, it was possible to identify the offsets between the human and exoskeleton joints for each subject. The protocol for calculating the offsets is outlined below.

1. Measure the distance from a fixed reflective marker on the back of the APO to the left and right exoskeleton joint centres.
2. Using the static pose data from this fixed marker, calculate the position in the ground frame of the left and right exoskeleton joint centres for each subject.
3. Using the reflective markers situated on the pelvis, coupled with a variation of the Harrington method (Harrington et al., 2007) for estimating the hip joint centre, calculate the locations of the left and right human hip joint centres in the ground frame.
4. Calculate the offset between the exoskeleton and human joint centres.

**Figure 2:**
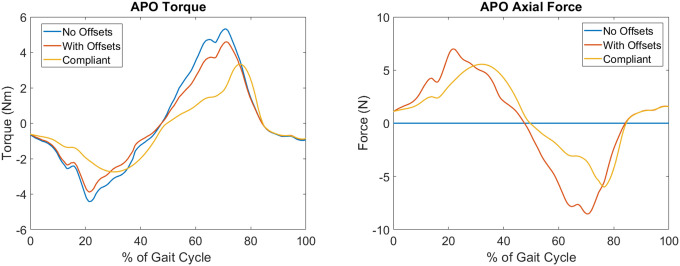
A graphical representation of the APO force models for a single gait cycle. The ideal force model (With Offsets) and compliant force model (Compliant) are shown relative to the measured APO torques (No Offsets). Left: the torque applied to the human femur body. Note the delayed onset of the peaks in the compliant force model. Right: the introduction of undesired interaction forces directed parallel to the thigh. These forces are introduced due to the presence of joint offsets.

The above steps were undertaken for all subjects using the corresponding static pose data. The offsets for each subject are summarised in Table 1. Once the offsets between the subjects and the APO were known, a model was derived following a similar strategy to previous works to decompose the torque generated by the APO in to an assistive torque applied to the human hip joint and an undesired interaction force which is applied parallel to the thigh (Schiele, 2008; Malosio et al., 2011). A sample APO torque trajectory is provided in Figure 2 which displays this decomposition for a single gait cycle.

**Table 1:** The static joint offsets between the hip joints of each subject and the APO joint centres.

Subject	Right hip offset (m)		Left hip offset (m)	
	x	y	x	y
S1	0.0818	0.0158	0.0817	0.0097
S2	0.0546	−0.0222	0.0621	−0.0288
S3	0.0847	0.0127	0.0843	0.0141
S4	0.0770	0.0057	0.0636	0.0105
S5	0.0929	0.0067	0.0648	0.0017
S6	0.0615	−0.0017	0.0584	0.0006
S7	0.0881	0.0209	0.1081	0.0213
S8	0.0753	0.0024	0.0580	0.0022

Note that the x axis is directed forward from the pelvis and the y axis is directed upwards.

### 2.5. APO Torque Transmission Models

Due to the presence of compliance in human-exoskeleton systems, largely due to flexible straps and soft biological tissues, power loss occurs between the torques generated by the exoskeleton and the torques experienced by the human subject. A relatively limited number of previous studies have investigated these interface dynamics in more detail, using a mix of kinematic and load sensing measurement devices to estimate the visco-elastic properties of the human-exoskeleton system (Schiele, 2008) or the relative timing and magnitude of power loss (Yandell et al., 2017).

In subsequent metabolic analyses of the APO in assistive mode, two models were used for the transmission of exoskeleton torques. Both models account for human-exoskeleton joint misalignment. The first model, hereafter referred to as the **ideal** torque transmission model, assumes 100% transfer of torque from the exoskeleton to the human. The second model, referred to as the **compliant** torque transmission model, assumes that the torque transmission is subject to absorption-return dynamics as observed by a recent study in to the interface dynamics of a soft exosuit (Yandell et al., 2017).

The compliant torque transmission model partitions the APO torque signal in to phases categorised as **loading** while the APO torques are increasing in magnitude and **unloading** while the torques are decreasing in magnitude. A percentage A of the power generated by the APO during loading phases is absorbed by the soft tissue in the system, and a percentage of this absorbed power, R, is returned during the next unloading phase. This results in a temporal offset between the peak applied torque of the ideal and compliant models. In Figure 2, a comparison between the ideal and compliant torque models is displayed for a single gait cycle. The absorption and return rates were chosen to be A=0.55 and R=0.75, respectively, to match the observations of Yandell et al. and allow for qualitative discussion on the compliant human-APO interface.

### 2.6. OpenSim Analyses

The processed data for each subject was divided in to 10 gait cycles per combination of walking context and assistance scenario. A range of analyses were then carried out using OpenSim tools in combination with the gait2392 and human/APO musculoskeletal models.

The first step was to scale the generic versions of these models to fit each subject using the static pose data and the **Scaling Tool**, which matched experimental markers placed on bony landmarks to the corresponding virtual markers placed on the model. The gait2392 model was scaled using static pose data from the NE assistance scenario, while the human/APO model was scaled using data from the ET assistance scenario. After scaling, the following sequence of analyses was carried out for each gait cycle:

- The **Inverse Kinematics Tool** was used to calculate joint angles given the marker trajectories.
- The **RRA Tool**, which accounts for dynamic inconsistency between the musculoskeletal model and the measured data (Delp et al., 2007), was used to produce dynamically consistent joint angles and a corrected model file from joint angles and ground reaction forces.
- The **Inverse Dynamics Tool** was used to calculate joint torques given the RRA-corrected joint angles and ground reaction forces.
- The **Analysis Tool** was used to calculate the position and velocity trajectory of the centre of mass of the model given the RRA-corrected kinematics.

**Figure 3:**
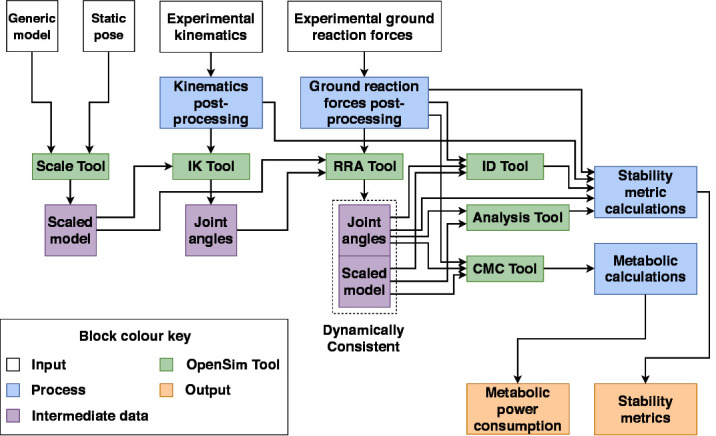
A schematic outlining the data collection and analysis pipeline.

Each of the dynamic analyses was performed twice for the EA assistance scenario; once using the ideal APO torque transmission model and again using the compliant model. The outputs of these analyses were used in the calculation of the stability metrics and metabolic energy consumption. A schematic of the overall data processing and analysis pipeline is provided in Figure 3.

### 2.7. Candidate Stability Metrics

A set of candidate stability metrics were chosen so as to cover a range of spatial, temporal and derived metrics. It is posited that those gait metrics which exhibit a strong invariance to changes in walking context or exoskeleton assistance scenario are good candidates for use as a measure of gait stability in variable walking conditions. The metrics and corresponding definitions were as follows:

- Step width was determined as the medial-lateral distance between the lateral malleolus markers at the heel strikes of consecutive steps.
- Step frequency was calculated as the inverse of the time between the heel strikes of consecutive steps.
- The hip range of motion ($\theta_{\text{hip-RoM}}$) was calculated by subtracting the maximum hip flexion joint angle from the minimum over the gait cycle.
- Hip peak to peak torques ($\tau_{\text{hip-pp}}$) were calculated by subtracting the maximum hip flexion joint torque from the minimum over the gait cycle.
- The CoM displacement was calculated by subtracting the maximum CoM position from the minimum over the gait cycle. This was calculated in both the vertical and medio-lateral directions, resulting in two distinct metrics: ${\text{CoM-V}}_{\text{disp}}$ and ${\text{CoM-ML}}_{\text{disp}}$, respectively.
- The CoP displacement was calculated by subtracting the maximum CoP position from the minimum over the stance phase period. This was calculated in both the anterior-posterior and medio-lateral directions, resulting in two distinct metrics: ${\text{CoP-AP}}_{\text{disp}}$ and ${\text{CoP-ML}}_{\text{disp}}$, respectively.
- The margin of stability was calculated as specified by Hof (Hof et al., 2005):

(2) $$\text{MoS}=|u_{max}-\left(x+\frac{v}{\omega_{0}}\right)|,$$

where $u_{max}$ is the boundary of the base of support, x is the centre of mass position, v is the centre of mass velocity, and $\omega_{0}$ is equal to:

(3) $$\omega_{0}=\sqrt{\frac{g}{l}},$$

with g denoting acceleration due to gravity and l being the distance from the pelvis ASIS to the lateral malleolus. The MoS was calculated in both the anterior-posterior and medio-laterial directions, resulting in two distinct metrics: MoS-AP and MoS-ML, respectively.

These metrics are summarised for reference in Table 2, which gives the names, units and notation for each metric.

**Table 2:** The direction, notation, and units of each metric.

Metric	Direction	Notation	Unit
Step width	N/A	N/A	cm
Step frequency	N/A	N/A	steps/min
Sagittal hip angle range of motion	N/A	$\theta_{\text{hip-RoM}}$	degrees
Sagittal peak to peak hip torque	N/A	$\tau_{\text{hip-pp}}$	Nm/kg
CoM displacement	Vertical	${\text{CoM-V}}_{\text{disp}}$	mm
CoM displacement	Medio-lateral	${\text{CoM-ML}}_{\text{disp}}$	mm
CoP displacement	Medio-lateral	${\text{CoP-ML}}_{\text{disp}}$	mm
CoP displacement	Anterior-posterior	${\text{CoP-AP}}_{\text{disp}}$	mm
Margin of stability	Medio-lateral	MoS-ML	mm
Margin of stability	Anterior-posterior	MoS-AP	mm

Note that stability was considered for the system as a whole, and therefore the net kinematic and ground reaction force data was used for metric calculations within the EA assistance scenario. The APO force models were not used to distinguish between the human and exoskeleton torque contributions in this case.

### 2.8. Modelling Metabolic Power Consumption

The calculation of metabolic power consumption followed a strategy used in other works (Uchida et al., 2016b; Dembia et al., 2017). The CMC algorithm (Thelen and Anderson, 2006) was run for each gait cycle, with a concurrent metabolics probe used to compute the instantaneous metabolic power consumption, $\dot{E}(t)$, for each muscle in the model. This probe computes the metabolic power consumption according to a muscle energetics model implemented by the OpenSim developers (Uchida et al., 2016a), which in turn is based on a previous model (Umberger et al., 2003).

The gait2392 model and our adapted human/APO musculoskeletal model both use Hill-type muscle models implemented by the OpenSim developers, which represent muscles as musculo-tendon units (MTUs) consisting of a tendon in series with a contractile muscle (Thelen, 2003). Each muscle is characterised by its maximum isometric force, optimal muscle fiber length, tendon slack length, maximum contraction velocity, and pennation angle. The values of these parameters are informed by previous studies (Wickiewicz et al., 1983; Friederich and Brand, 1990; Delp et al., 1990b; Anderson and Pandy, 1999b).

Once calculated, the instantaneous metabolic power consumption of each muscle was integrated over the gait cycle and divided by total subject mass (m) and gait cycle length (in seconds) to produce the normalised, average metabolic power consumption, as follows:

(4) $${\bar{P}}_{\text{avg}}=\frac{1}{m\left(t_{1}-t_{0}\right)}\int_{t_{0}}^{t_{1}}\;\overset{˙}{E}\left(t\right)\text{ d}t.$$

Normalised averaged metabolic power consumption was calculated both using the ideal APO torque transmission model and, separately, the compliant model.

### 2.9. Statistical Analysis

The mean and SD of each stability metric was averaged over all recorded gait cycles and all subjects, for each combination of walking context and assistance scenario. Therefore, the number of samples for each combination of stability metric, walking context and assistance scenario was 70 for most metrics (10 gait cycles × 7 subjects^1^). An exception was the step width metric, which relies on pairs of adjacent gait cycles for its computation, and therefore had a sample size of 63 (9 pairs of adjacent gait cycles × 7 subjects).

The mean and SD of the normalised average metabolic power consumption was calculated in the same way as outlined above for each muscle in the musculoskeletal model, however only half of the recorded gait cycles were used for each subject to reduce the time taken to perform the simulations. Consequently, the number of samples for each combination of muscle, walking context and assistance scenario was 35 (5 gait cycles × 7 subjects).

To investigate the effects of the exoskeleton assistance and the walking context on the stability metrics, a two-way ANOVA was used. For the post-hoc analysis, the MATLAB multiple comparison procedure “multcompare” was used with the comparison type based on Tukey’s honestly significant difference criterion. The statistical significance level was set at α=0.05.

For combinations of walking context and assistance scenario which demonstrated a significant difference in the mean of a metric, the effect size was measured by computing the absolute value^2^ of Cohen’s d. These values were then averaged to produce a quantitative measure of invariance for each metric relative to changes in assistance level, changes in walking context, and overall. Qualitative analysis of the effect size of each metric was undertaken according to typical cutoffs for the value of Cohen’s d (Cohen, 1988; Sawilowsky, 2009), which are provided for reference in Table 3.

**Table 3:** A mapping from qualitative descriptions of effect size to the corresponding range of Cohen’s d.

Effect size	Cohen’s d range
Very small	0.01 ≤ *d* < 0.20
Small	0.20 ≤ *d* < 0.50
Medium	0.50 ≤ *d* < 0.80
Large	0.80 ≤ *d* < 1.20
Very large	1.20 ≤ *d* < 2.00
Huge	*d* ≥ 2.00

The two-way ANOVA and multiple comparison procedure was repeated with the same statistical significance level to investigate changes in normalised average metabolic power consumption for a subset of muscles crossing the hip, knee and ankle joints. The included muscles were as follows: the adductor brevis, adductor longus, adductor magnus, psoas, gluteus maximus, biceps femoris, rectus femoris, vastus medialis, medial gastrocnemius and soleus. For reference these muscles and their main actions are listed in Table 4.

**Table 4:** The muscles for which a two-way ANOVA analysis was carried out, along with their main actions.

Muscle	Actions
Adductor brevis	Hip adduction
Adductor longus	Hip adduction, hip flexion
Adductor magnus	Hip adduction, hip flexion, hip extension
Psoas	Hip flexion
Gluteus maximus	Hip extension, hip rotation
Biceps femoris long head	Knee flexion, hip extension
Rectus femoris	Knee extension, hip flexion
Vastus medialis	Knee extension
Medial gastrocnemius	Ankle plantarflexion, knee flexion
Soleus	Ankle plantarflexion

In order to directly compare the effect of active exoskeleton assistance between different contexts, a one-way ANOVA was performed for each context over all assistance levels and for each muscle. Within each context, the muscles which had significantly different average metabolic power consumption when in active-ideal assistance mode (EA-I) or active-compliant assistive mode (EA-C) compared to transparent mode (ET) were identified. The relative change in metabolic power consumption going from transparent mode to active mode was then calculated as a percentage in order to quantify the effectiveness of the exoskeleton assistance.

## 3. Results

The anthropometric measurements and calculated walking velocities for each subject are presented in Table 5.

**Table 5:** The subjects’ anthropometric features and walking velocities. ©IEEE 2017

Subject	Height (m)	Weight (kg)	Walking velocity (m/s)		
			BW	FW	SW
S1	1.84	76.4	0.95	1.14	0.76
S2	1.79	67.1	0.95	1.14	0.76
S3	1.74	58.8	0.94	1.13	0.75
S4	1.76	77.2	0.94	1.13	0.75
S5	1.88	83.0	0.97	1.18	0.78
S6	1.80	61.4	0.96	1.15	0.77
S7	1.77	66.6	0.97	1.16	0.78
S8	1.80	75.8	0.95	1.14	0.76

Running the RRA tool for all the data sets generated residual forces and moments, which are applied to the pelvis in simulation to account for the dynamic inconsistency between the dynamic model and the recorded data. These residuals should be low to ensure accurate simulations. All of the average residual forces measured during our simulations were less than the thresholds specified by the OpenSim developers (see Table 6).

**Table 6:** RRA residuals in OpenSim. ©IEEE 2017

Quantity	Value	OpenSim Benchmark
RMS Residual force (N)	7.1±3.4	<10
Peak Residual force (N)	17.9±7.6	<25
RMS Residual moment (Nm)	7.3±4.0	<50
Peak Residual moment (Nm)	16.9±8.7	<75

### 3.1. Gait Metric Invariance

**Figure 4:**
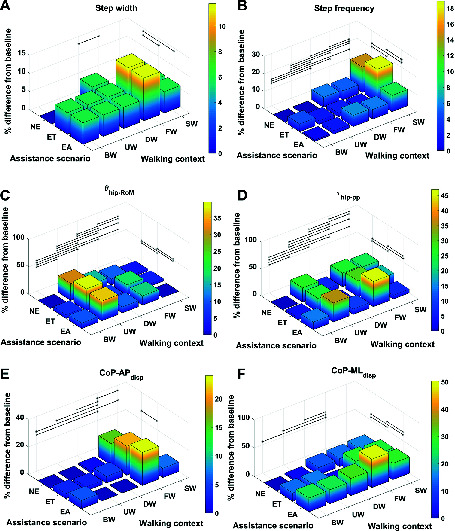
Percentage difference from baseline, categorised by walking context and assistance scenario, for **(A)** step width, **(B)** step frequency, **(C)**$\theta_{\text{hip-RoM}}$, **(D)**$\tau_{\text{hip-pp}}$, **(E)**${\text{CoP-AP}}_{\text{disp}}$ and **(F)**${\text{CoP-ML}}_{\text{disp}}$. Black lines represent significant differences. ©IEEE 2017

**Figure 5:**
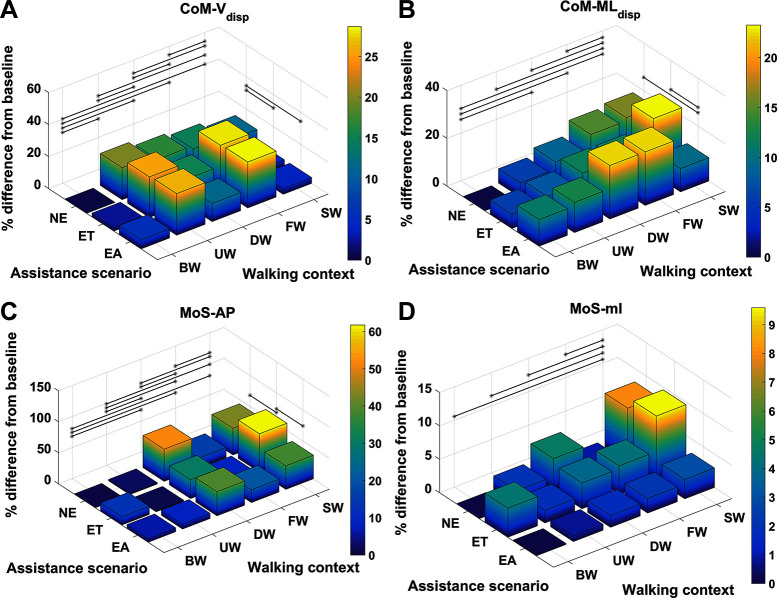
Percentage difference from baseline, categorised by walking context and assistance scenario, for **(A)**${\text{CoM-V}}_{\text{disp}}$, **(B)**${\text{CoM-ML}}_{\text{disp}}$, **(C)**MoS-AP and **(D)**MoS-ML. Black lines represent significant differences. ©IEEE 2017

For each metric and for every context and assistance scenario the percentage difference from the baseline condition (no exoskeleton assistance and walking at baseline speed) is shown in Figures 4 and 5. Additionally, the mean and SD values for every context and assistance scenario combination are detailed in the Table S1 (Data Sheet S1).

**Figure 6:**
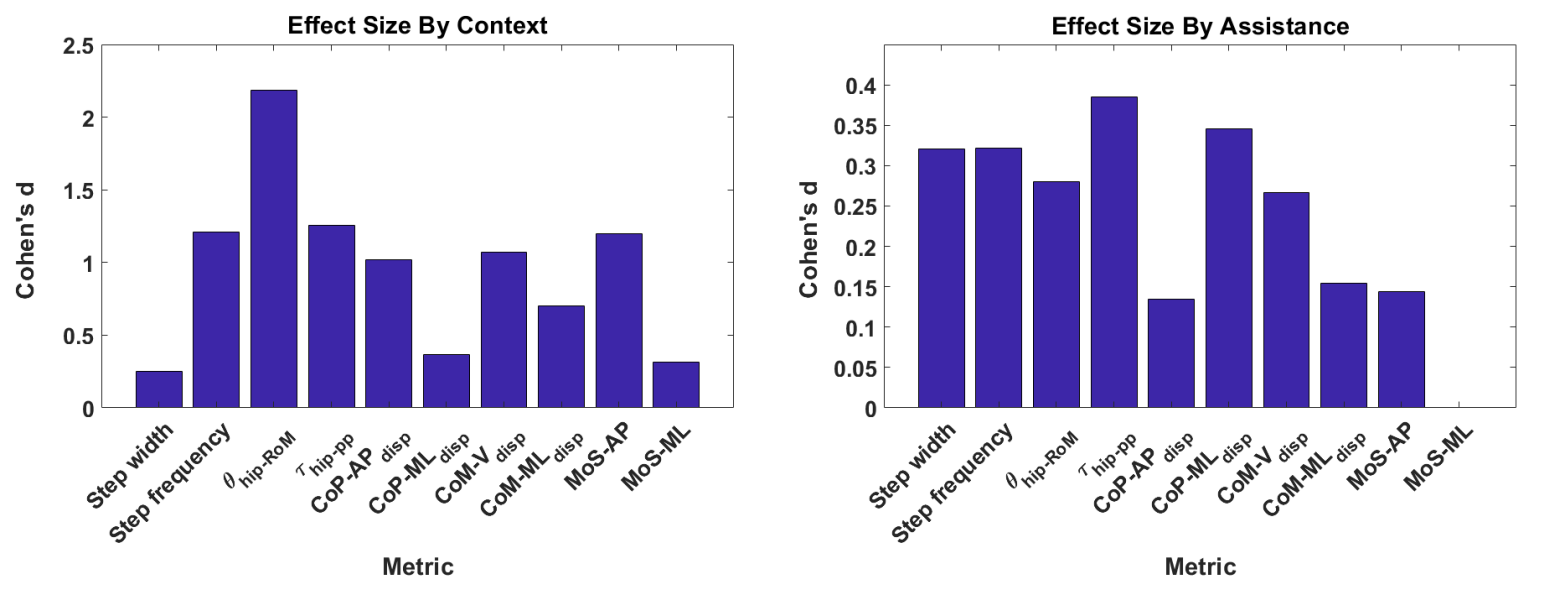
The effect sizes for each gait metric, averaged over **(A)** context and **(B)** assistance. Note that no significant differences due to changes in assistance level were observed for the MoS-ML metric. ©IEEE 2017

The effect sizes for each metric, averaged separately by walking context and by assistance scenario, are displayed in Figure 6. Comparing the effect sizes averaged over walking contexts, it is demonstrated that step width, ${\text{CoP-ML}}_{\text{disp}}$, and MoS-ML have the lowest average effect size. These metrics have Cohen’s d values that indicate between small and medium effect sizes. The $\theta_{\text{hip-RoM}}$ metric exhibits a Cohen’s d of greater than 2.0, which implies a huge effect size. All other metrics show effect sizes of large or very large. This analysis implies that, relative to changes in walking context, the step width, ${\text{CoP-ML}}_{\text{disp}}$, and MoS-ML metrics demonstrate the highest level of invariance.

Comparing the effect sizes averaged over assistance scenario, the metrics which exhibit the lowest average effect size are ${\text{CoP-AP}}_{\text{disp}}$, ${\text{CoM-ML}}_{\text{disp}}$ and, similarly to the context results, MoS-ML. These metrics exhibit a Cohen’s d of below 0.2, which indicates a very small effect size. All other metrics exhibit a small effect size. Compared to the results for walking context, the effect of changing assistance scenario is in general less than for changing walking context. This analysis implies that the ${\text{CoP-AP}}_{\text{disp}}$, ${\text{CoM-ML}}_{\text{disp}}$ and MoS-ML metrics demonstrate the highest level of invariance relative to changes in the assistance scenario.

**Figure 7:**
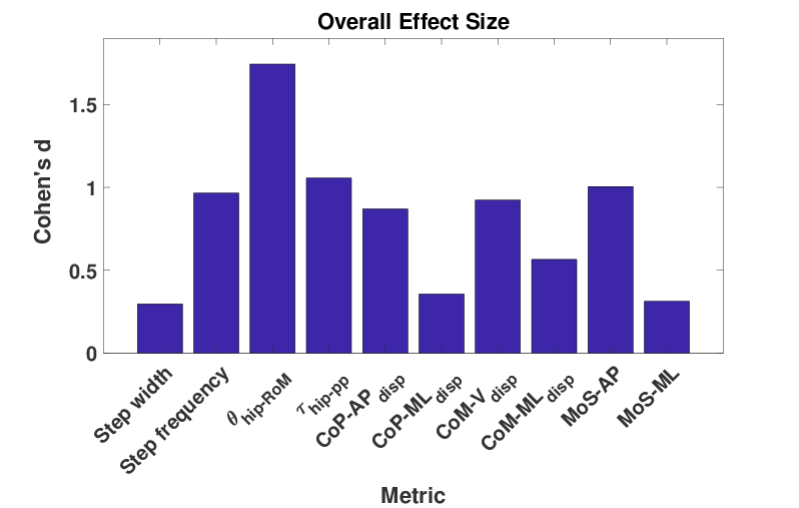
The effect sizes for each gait metric, averaged over all combinations of walking context and assistance scenarios. ©IEEE 2017

In Figure 7, the effect size is shown for each metric, averaged over both walking context and assistance level simultaneously. From these results, we see that step width, ${\text{CoP-ML}}_{\text{disp}}$ and MoS-ML are the metrics which exhibit the lowest effect size, with values of Cohen’s d between 0.2 and 0.5 corresponding to a medium effect. Analysing the remaining metrics, we see that the ${\text{CoM-ML}}_{\text{disp}}$ metric exhibits a medium effect size, the $\theta_{\text{hip-RoM}}$ metric exhibits a very large effect size, and all remaining metrics exhibit large effect sizes. From this analysis we conclude that overall, taking in to account both changes in walking context and assistance scenario, the three metrics which exhibit the most invariance are step width, ${\text{CoP-ML}}_{\text{disp}}$ and MoS-ML.

### 3.2. Metabolic Power Consumption

**Figure 8:**
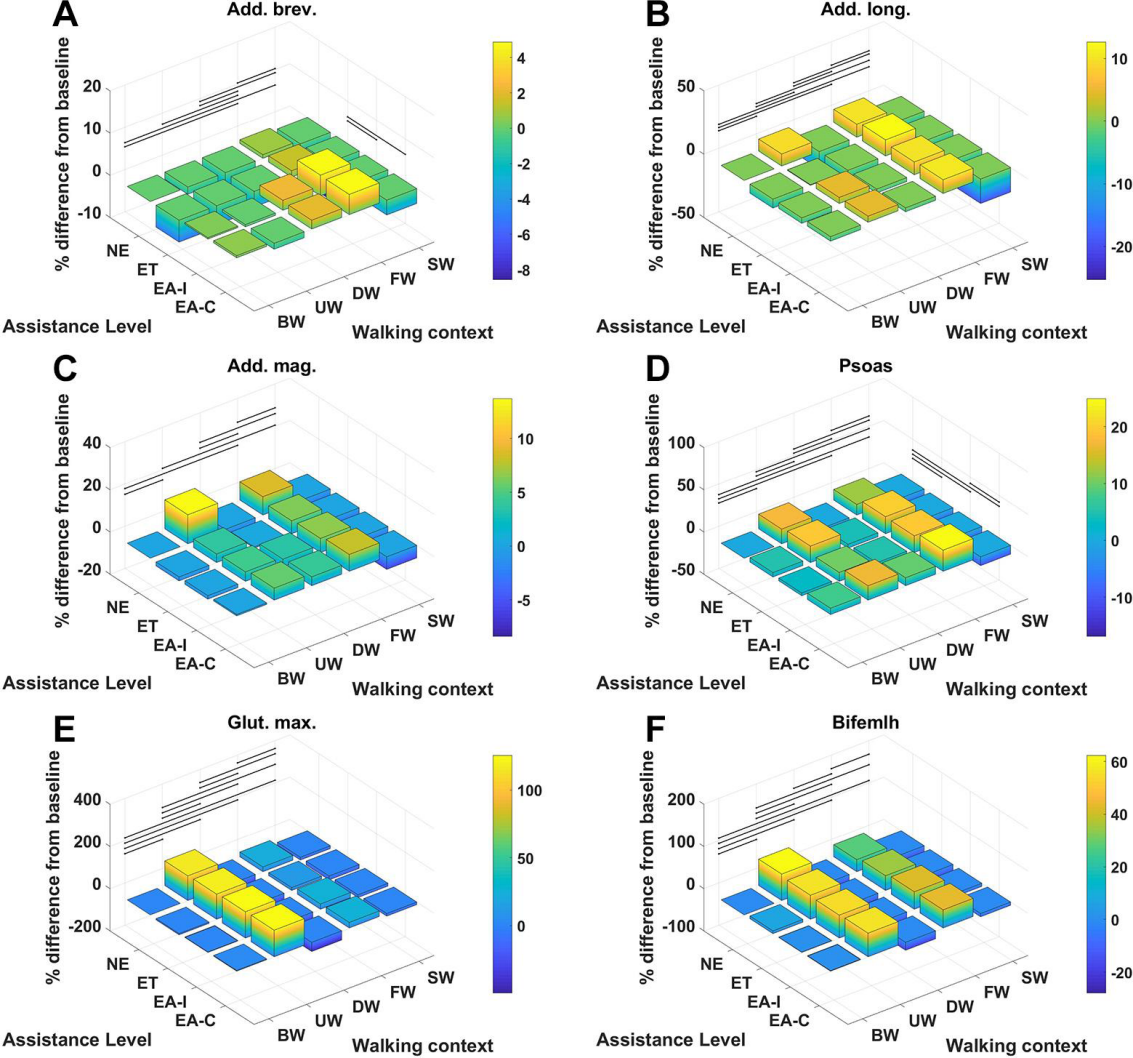
The % difference in average metabolic power consumption of the **(A)** adductor brevis, **(B)** adductor longus, **(C)** adductor magnus, **(D)** psoas, **(E)** gluteus maximus and **(F)** biceps femoris long head muscles.

**Figure 9:**
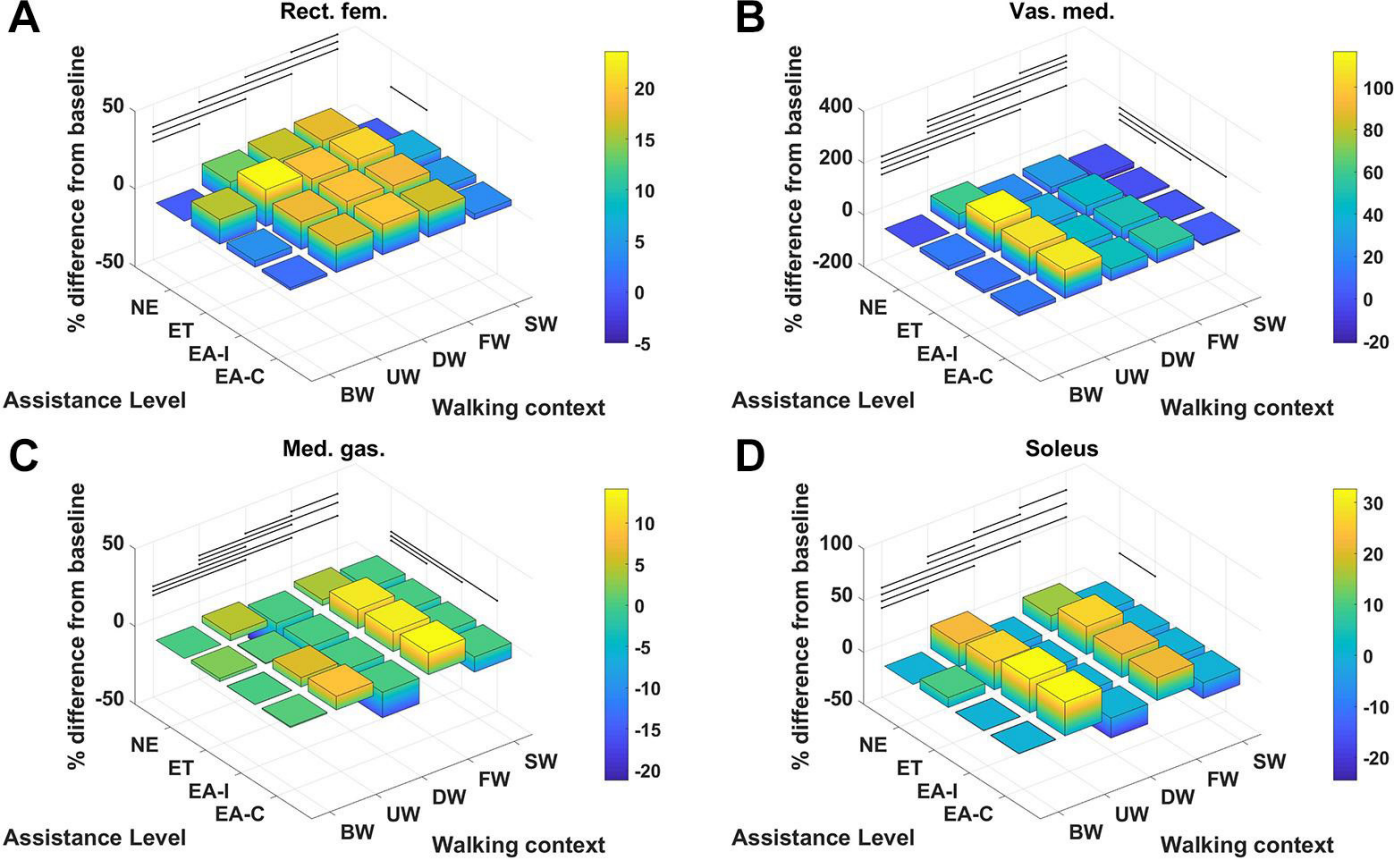
The % difference in average metabolic power consumption of the **(A)** rectus femoris, **(B)** vastus medialis, **(C)** medial gastrocnemius and **(D)** soleus muscles.

For each muscle in Table 4 the percentage difference in average metabolic power consumption from the baseline condition (no exoskeleton assistance and walking at baseline speed) as a function of walking context and assistance level is demonstrated in Figures 8 and 9. Additionally, the raw mean and SD values for every context and assistance scenario combination are detailed in Table S2 (Data Sheet S1). For analysis purposes the data from the two APO force models are presented as distinct assistance levels.

**Figure 10:**
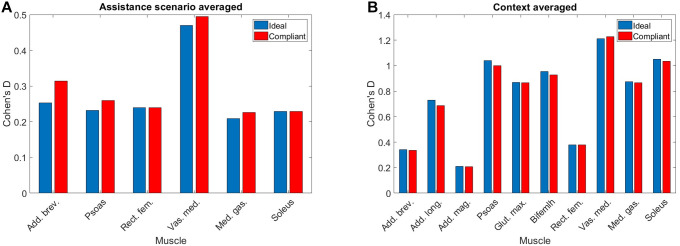
The values of Cohen‘s d for each muscle in our subset averaged by **(A)** assistance and **(B)** context. Note that absence of a muscle denotes that no significant differences were observed.

Given the cases where significant differences in the average metabolic power consumption were observed, the effect size was averaged along assistance and context. The results are presented in Figure 10. Notably, the change in assistance had no significant effect on four of the muscles, namely: the adductor longus, adductor magnus, gluteus maximus or biceps femoris long head. Changes in walking context introduced at least some significant differences to all muscles in the subset.

In general, the observed effect of assistance level on the average metabolic power consumption of the muscles was small, with all effect sizes lying within the range 0.2≤0.5 regardless of whether the ideal or compliant APO force model was used. In each of the muscles which demonstrated a significant effect, the compliant APO force model resulted in a slightly more pronounced or equivalent effect size when compared to the ideal force model.

In contrast, the muscles we considered experienced a wider range of effect sizes due to changing walking context. The psoas, gluteus maximus, biceps femoris long head, medial gastrocnemius and soleus muscles all exhibited large effect sizes, with a Cohen’s d of greater than 0.8 but less than 1.2. The vastus medialis was the muscle which was most affected by changes in walking context, exhibiting a very large effect size. The adductor longus experienced a medium effect size and the adductor brevis, adductor magnus, and rectus femoris all experienced small effect sizes. Similar to the assistance-averaged case, use of the ideal or compliant force models did not alter the classification of the effect size for any muscle.

From Figures 8 and 9 we can see that only one muscle from our subset shows a significant difference in metabolic energy consumption on average over all contexts due to the transition from transparent mode to active assistance, namely the adductor brevis muscle. Five muscles, the psoas, rectus femoris, vastus medialis, medial gastrocnmeius and soleus show significant differences between the NE and ET assistance levels, which implies the change in metabolic energy consumption of these muscles is attributable to the increased physical load of wearing the exoskeleton rather than the presence of active assistance itself. One muscle, the psoas, shows a significant difference between the APO force models.

**Figure 11:**
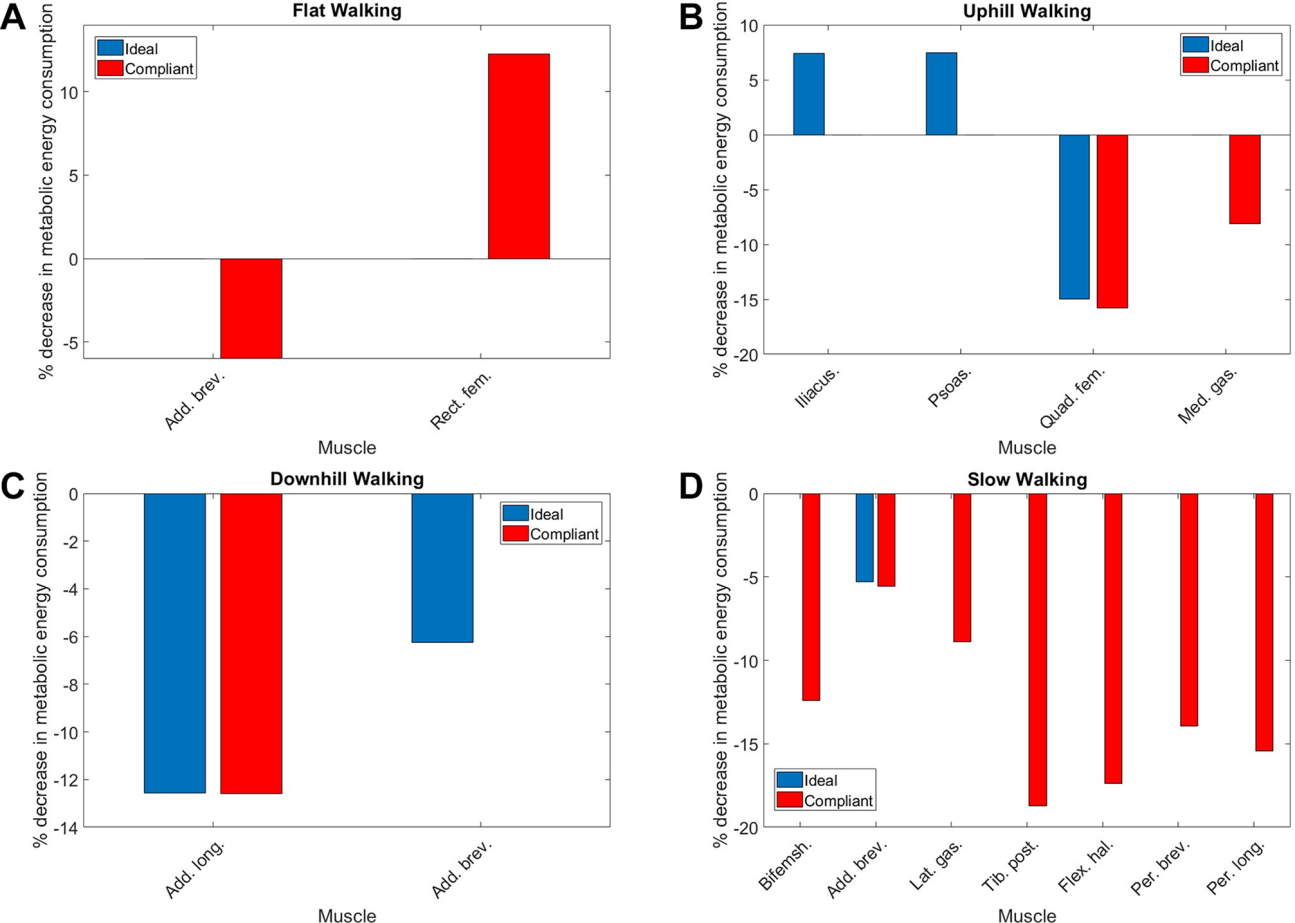
The percentage change in average normalised metabolic energy consumption of muscles which show a significant change between the ET and EA assistance modes during **(A)** flat walking, **(B)** uphill walking, **(C)** downhill walking and **(D)** slow walking. Note that red bars correspond to the compliant force model whereas blue bars correspond to the ideal force model. Absence of data denotes that no significant differences were observed in this case.

**Figure 12:**
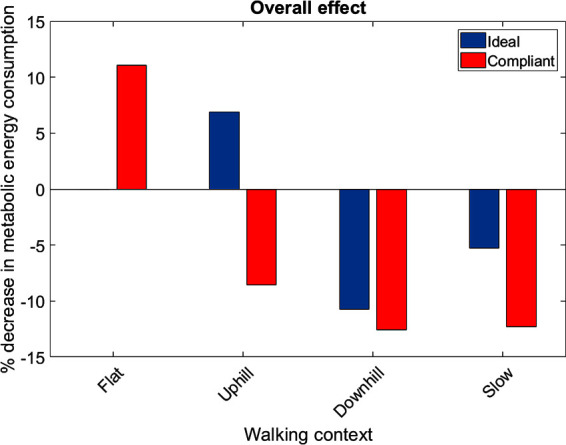
The overall metabolic effect of exoskeleton assistance in each walking condition, for both the ideal APO force model (blue) and the compliant APO force model (red). Absence of data denotes that no significant differences were observed in this case.

The muscles from the full set which were identified via the context-specific one-way ANOVAS to experience a significant change in metabolic energy consumption between the ET and EA assistance levels are listed by context in Table 7. Note that significant differences were seen for the uphill walking, downhill walking and slow walking contexts for both force models. Significant differences were seen in the flat walking context, but only when the compliant model was used. No significant differences were seen in the fast walking context. The relative change in metabolic power consumption is shown for the muscles in these contexts in Figure 11. The overall effect, calculated from total significant metabolic energy change in each context, is presented in Figure 12. We see from these results that the active exoskeleton assistance has a positive effect in the BW scenario, but only when the compliant APO force model is used. There is a disagreement in the UW context, where the ideal APO force model predicts a net assistance of approximately 8%, whereas the compliant force model predicts that human metabolic energy consumption is increased. The ideal force model predicts that the iliacus and psoas muscles are assisted; the main actions of these muscles is to assist hip flexion. Meanwhile, both force models agree that the active exoskeleton assistance is detrimental in the SW and DW scenarios, where human metabolic energy consumption is seen to increase by between approximately 5% and 12% depending on the walking context and which force model is used.

**Table 7:** The muscles which are significantly affected by exoskeleton assistance for each context.

Baseline walking	Uphill walking	Downhill walking	Slow walking
Adductor brevis	Iliacus	Adductor longus	Adductor brevis
Rectus femoris	Psoas	Tibialis posterior	Adductor brevis
	Quadratus femoris		Biceps femoris long head
	Medial gastrocnemius		Lateral gastrocnemius
			Flexor hallucis longus
			Peroneus brevis
			Peroneus longus

## 4. Discussion

### 4.1. Stability Metrics

It is well known that walking speed is a cause of gait variability for kinematic, kinetic, and ${\text{CoM}}_{\text{disp}}$ metrics (Winter, 1984; Stansfield et al., 2001; Orendurff et al., 2004) and the results from this study also demonstrate the same findings. This study demonstrates that step frequency increases due to speed and anecdotally suggests that step length increases as well (step length was not included in the analysis due to unavailability of complete consecutive step data for some of the participants due to cross-talk on the force plates). The step width metric was demonstrated to be invariant with only one significant difference with a small effect size between walking downhill and walking at a fast speed. Walking speed causes variation for the ${\text{CoP-AP}}_{\text{disp}}$ and the ${\text{CoP-ML}}_{\text{disp}}$ metrics, however, the effect on ${\text{CoP-ML}}_{\text{disp}}$ is small and only between the fast and baseline walking speed contexts. In addition, walking speed affects the MoS-AP and MoS-ML metrics, however there is only a small effect in the medio-lateral direction between the slow and baseline walking speed contexts. It is a logical result that walking speed has a greater effect on the metrics measured in the anterior posterior direction compared to the medial lateral direction because walking speed is a change of direction mainly in the anterior posterior direction.

The effect of walking up and down an incline has previously been demonstrated to have significant kinematic and kinetic changes (Lay et al., 2006) and the results from this study support this result. In addition to the effects on kinematics and kinetics, the results from this study indicate that walking on an incline affects the ${\text{CoM-V}}_{\text{disp}}$ metric and that walking down an incline affects the step frequency, ${\text{CoM-V}}_{\text{disp}}$, ${\text{CoM-ML}}_{\text{disp}}$, and MoS-AP metrics. The increase in step frequency is expected due to a shorter step length being taken. The effects on the CoM vertical displacement are also expected due to the change in height caused by the slope. The effect on the MoS-AP metric is expected because this measures the stability in the backwards direction and it is clear that when the torso is tilted forwards the MoS-AP values will increase. Neither walking up or down an incline had a significant effect on the MoS-ML .

This study demonstrates there are significant differences between the $\theta_{\text{hip-RoM}}$ metric caused by context and assistance scenario changes. This result aligns with the work by d’Elia et al. (d'Elia et al., 2017), and similarly their claim that the differences between the assistance scenario $\theta_{\text{hip-RoM}}$ results are within the natural variation of gait is also applicable. There was a significant increase in the $\tau_{\text{hip-pp}}$ metric from the ET to the EA scenario. This result suggests a disagreement with a study by Lewis and Ferris (Lewis and Ferris, 2011), which found that the net torques did not change between walking with an exoskeleton in passive mode and with it in assistive mode. The result from our study suggests that torque from the exoskeleton is not entirely being transferred to the individual, which is quite probable due to the non-rigid attachments of the exoskeleton. There is a significant increase in the ${\text{CoP-ML}}_{\text{disp}}$ metric from the NE and the ET and EA scenarios, which can be attributed to the extra weight of the exoskeleton laterally located to the participant. One consideration for the above findings is that the effect sizes for all the metrics for the assistance scenarios were between small and medium, which suggests the differences are small between the assistance scenarios. This is in contrast to the context effect sizes, where 6 out of the 10 metrics had large or greater effect sizes.

All gait metrics exhibited some significant differences due to the changes in walking context and assistance scenario. After factoring in the effect sizes the most invariant metrics were shown to be step width, ${\text{CoP-ML}}_{\text{disp}}$, and MoS-ML. All three metrics have been demonstrated to be associated with stability (Hof et al., 2005; Kuo and Donelan, 2010; Svoboda et al., 2016), and therefore it is intuitive that they remain fairly constant despite changes in walking context and the application of small constant perturbations. The MoS metric is most suitable for use in a control paradigm because it can be calculated at any given time during a gait cycle and therefore enables a significantly more responsive controller. As a source of further work, our investigation of stability metrics could be repeated with patients to determine how the invariance properties of the metrics are affected by gait pathologies.

### 4.2. Metabolic Power Consumption

In general, we observed that applying exoskeleton assistance had significantly less effect on metabolic energy consumption than changes in walking context. However, this analysis was limited by the fact that while there were three assistance scenarios, only one of these scenarios explored active assistance, and therefore comparison between different magnitudes of active assistance was not explored. A source of further work could be to collect data using a wider range of virtual stiffness levels, which would allow for an analysis of how the metabolic effect of active assistance varies with assistance magnitude. It should be noted that the motor torques commanded to the APO during active assistance trials were already close to the torque limitations in place on the device.

The relative effect of applying exoskeleton assistance was most pronounced in the flat walking, uphill walking, downhill walking and slow walking scenarios. In the latter two of these scenarios, both the ideal and compliant APO force models predicted increased metabolic cost. The compliant model predicted that flat walking benefited from assistance, while the ideal model predicted that uphill walking benefited from assistance. Anecdotally, this result agrees with feedback from subjects following data collection (e.g., the exoskeleton assistance was most beneficial when walking uphill). The negative effect of the exoskeleton when walking at slow speed may be a result of the choice of control algorithm. As discussed in Section 2.1, the control algorithm used is based on adaptive oscillators, which requires synchronisation to input joint angles. Therefore, the decrease in performance in the slow walking context may have been due to a suboptimal synchronisation. However, during walking trials, time was allowed both for subject familiarisation with the new context and for APO controller synchronisation.

A limiting factor of our study is that the adaptive oscillator control sceheme was the only exoskeleton controller tested. A source of further work could be to apply or develop additional control paradigms, so that their relative performance over different contexts can be analysed. Additionally, external measurement devices such as calorimetry systems, which have recently been used for investigations in to metabolic cost reductions of soft exosuits (Quinlivan et al., 2017), could be helpful in directly quantifying changes in net metabolic activity in future experiments, reinforcing our simulation framework.

It should be noted that the implementation of a compliant APO force model was intended largely as a point of qualitative comparison between the results from the ideal APO force model. Indeed, several differences between the experimental setup used for this work and the work on interface dynamics (Yandell et al., 2017) could lead to differing compliant behaviour in our case. However, these results do suggest qualitatively that including compliance in the system, as certainly is the case for contact between exoskeletons and soft straps or human tissue, can cause marked differences in the effectiveness of exoskeleton assistance. Further work is needed to implement control algorithms which can account for human-exoskeleton interface dynamics in real-time.

Overall, our results quantify the effect that varying walking context has on the effectiveness of active exoskeleton assistance. If exoskeletons are to be applied in real-world settings where subjects may frequently adapt their walking speed or incline, they must be able to rely on adaptive control algorithms which can account for these changes in walking context. Failure to do so can result in increased, rather than decreased, metabolic energy costs, as shown by our analysis. Techniques based on musculoskeletal modelling over various walking conditions can be useful and non-invasive tools for testing how well exoskeleton control algorithms perform in this regard.

## Ethics Statement

To carry out the experiments approval from the School of Informatics' Ethics Panel was received. Eight participants were recruited to undertake data collection after providing written informed consent.

## Author Contributions

DG and GH jointly created the human/APO model, devised the data collection protocol and carried out the experiments. DG implemented the batch OpenSim analysis pipeline and the metabolics calculations. GH implemented the post-processing pipeline and the gait metric calculations. SV provided continuous supervision and critical feedback on methods and results. All authors contributed to the drafting of the article.

## Conflict of Interest Statement

The authors declare that this research was conducted in the absence of any commercial or financial relationships that could be construed as a potential conflict of interest.

## Footnotes

1 The data from one subject had to be discarded due to an issue with the force plate calibration.

2 The decision to take the absolute value was motivated by an interest in the magnitude of an effect rather than its direction.
