# Supplementary material for: Effectively Quantifying the Performance of Lower-Limb Exoskeletons Over a Range of Walking Conditions
Source: Front Robot AI. 2018 Jun 27;5:61. doi: 10.3389/frobt.2018.00061 (PMC7904313; doi:10.3389/frobt.2018.00061)
Supplement: Supplementary file 1 [file DataSheet1.PDF]

Supplementary Material: Effectively quantifying the performance of lower-limb exoskeletons over a range of walking conditions

Daniel Gordon [1,*], Graham Henderson [1] and Sethu Vijayakumar [1]

*Correspondence:
Daniel Gordon
daniel.gordon@ed.ac.uk

1 SUPPLEMENTARY TABLES AND FIGURES

Table 1. Mean ±SD of metrics categorised by walking context and assistance scenario.

AS	Step width (cm)					Step frequency (steps/min)				
	BW	UW	DW	FW	SW	BW	UW	DW	FW	SW
NE	17.3 ± 4.1	16.8 ± 3.5	18.5 ± 5.1	16.8 ± 4.5	15.9 ± 4.4	103.4 ± 8.7	103.5 ± 9.8	109.4 ± 9.0	108.8 ± 8.5	88.3 ± 6.7
ET	16.2 ± 3.5	15.9 ± 2.2	16.2 ± 3.2	15.9 ± 3.6	15.9 ± 2.5	98.7 ± 14.8	101.3 ± 6.9	105.7 ± 6.5	104.9 ± 12.3	83.9 ± 8.8
EA	15.8 ± 3.1	15.8 ± 3.2	16.3 ± 3.2	15.8 ± 3.8	16.5 ± 3.6	104.3 ± 5.3	105.4 ± 6.7	110.1 ± 6.9	109.2 ± 12.9	93.7 ± 7.5
AS	$\theta_{\text{hip-RoM}}$(Sagittal hip angles RoM (°))					$\tau_{\text{hip-pp}}$(Sagittal peak to peak hip torques (Nm/kg))				
	BW	UW	DW	FW	SW	BW	UW	DW	FW	SW
NE	36.0 ± 3.0	47.2 ± 3.8	30.4 ± 3.6	40.0 ± 3.2	34.7 ± 3.7	0.98 ± 0.2	1.24 ± 0.2	0.91 ± 0.2	1.23 ± 0.2	0.76 ± 0.2
ET	38.4 ± 2.7	50.3 ± 2.7	33.3 ± 3.8	42.7 ± 2.8	37.6 ± 3.5	1.02 ± 0.2	1.23 ± 0.2	0.97 ± 0.1	1.27 ± 0.1	0.80 ± 0.2
EA	39.6 ± 3.6	49.0 ± 3.0	32.0 ± 3.2	42.2 ± 3.1	36.0 ± 5.2	1.12 ± 0.1	1.33 ± 0.1	1.08 ± 0.2	1.44 ± 0.3	0.91 ± 0.2
AS	CoP-AP$_{\text{disp}}$(CoP anterior posterior displacement (mm))					CoP-ML$_{\text{disp}}$(CoP medial lateral displacement (mm))				
	BW	UW	DW	FW	SW	BW	UW	DW	FW	SW
NE	419.1 ± 52.2	420.2 ± 56.8	413.4 ± 57.7	484.9 ± 83.7	403.3 ± 50.3	32.2 ± 14.6	31.3 ± 9.6	28.1 ± 13.6	36.8 ± 16.8	37.5 ± 20.3
ET	434.7 ± 45.8	431.3 ± 46.7	439.6 ± 81.8	508.0 ± 51.6	427.5 ± 49.2	37.0 ± 16.5	37.2 ± 13.8	31.5 ± 14.9	41.2 ± 16.9	40.7 ± 28.5
EA	444.9 ± 65.4	419.2 ± 55.1	418.1 ± 79.9	519.9 ± 101.5	390.5 ± 51.4	40.1 ± 18.2	40.2 ± 14.8	42.1 ± 18.2	48.4 ± 24.2	41.5 ± 18.4
AS	CoM-V$_{\text{disp}}$(CoM vertical displacement (mm))					CoM-ML$_{\text{disp}}$(CoM medial lateral displacement (mm))				
	BW	UW	DW	FW	SW	BW	UW	DW	FW	SW
NE	30.0 ± 4.9	36.0 ± 6.0	35.0 ± 6.0	34.4 ± 4.0	26.6 ± 4.1	58.5 ± 19.7	55.0 ± 13.6	53.2 ± 19.3	49.8 ± 17.6	68.0 ± 23.2
ET	30.7 ± 4.8	37.6 ± 4.7	34.5 ± 5.9	38.4 ± 5.8	28.9 ± 5.1	55.0 ± 18.8	54.0 ± 12.2	51.6 ± 16.7	51.6 ± 16.0	72.1 ± 18.5
EA	31.7 ± 4.1	37.8 ± 5.0	33.4 ± 4.6	38.6 ± 5.7	28.9 ± 4.0	52.0 ± 13.7	51.1 ± 12.2	45.4 ± 14.3	45.4 ± 12.6	64.2 ± 18.7
AS	MoS-AP (Margins of stability - anterior posterior (mm))					MOS-ML (Margins of stability - medial lateral (mm))				
	BW	UW	DW	FW	SW	BW	UW	DW	FW	SW
NE	104.0 ± 36.1	101.8 ± 46.9	157.8 ± 45.4	120.8 ± 46.1	59.3 ± 36.3	68.8 ± 16.9	70.2 ± 18.6	72.05 ± 17.4	69.5 ± 20.6	63.3 ± 21.7
ET	90.2 ± 34.8	102.3 ± 39.6	135.6 ± 32.6	112.3 ± 25.6	39.8 ± 30.8	71.8 ± 17.3	70.1 ± 17.2	66.16 ± 15.3	71.75 ± 18.6	62.2 ± 18.6
EA	97.5 ± 25.7	112.5 ± 40.8	144.5 ± 41.9	125.5 ± 36.5	64.4 ± 30.1	68.7 ± 18.7	69.4 ± 15.8	70.0 ± 21.4	70.3 ± 18.5	66.8 ± 16.2

Table 2. Mean ±SD of average metabolic power consumption of muscles, categorised by walking context and assistance scenario. Units are in milli-Watts per kilogram (mW/kg).

AS	Adductor brevis (mW/kg)					Adductor longus (mW/kg)				
	BW	UW	DW	FW	SW	BW	UW	DW	FW	SW
NE	17.2 ± 2.7	16.9 ± 2.1	16.4 ± 2.2	17.3 ± 2.8	16.2 ± 1.9	47.4 ± 13.7	51.8 ± 13.8	40.9 ± 11.7	52.2 ± 13.5	37.1 ± 8.8
ET	16.3 ± 2	16.4 ± 2.3	16.6 ± 2.2	17.4 ± 2.8	15.7 ± 1.8	44 ± 10.5	47.1 ± 12.1	40.8 ± 10.3	53.4 ± 15.6	35.4 ± 7.9
EA	17.3 ± 2.6	17 ± 2.7	17.5 ± 3.1	18 ± 3.1	16.6 ± 2.2	44.8 ± 13.6	48.2 ± 13.4	45.5 ± 13.3	51.1 ± 17.6	37.4 ± 8.3
AS	Adductor magnus (mW/kg)					Psoas (mW/kg)				
	BW	UW	DW	FW	SW	BW	UW	DW	FW	SW
NE	14.2 ± 6.1	16.1 ± 9.3	13.4 ± 5.3	15.4 ± 7.5	13 ± 4.8	194.4 ± 40.8	228 ± 38.8	178.6 ± 35	218.6 ± 37.5	161.6 ± 27
ET	13.9 ± 6.1	14.7 ± 7.9	14.2 ± 7.3	15 ± 7.6	13 ± 5.3	204.3 ± 32.5	232 ± 30.8	203.1 ± 38.5	233.5 ± 34.9	169.3 ± 27
EA	14 ± 6	14.9 ± 7.9	14.7 ± 8	15.1 ± 7.7	13.3 ± 5.2	198.1 ± 35.2	213.4 ± 32.7	202.2 ± 37	229.9 ± 53.7	166.2 ± 33.8
AS	Gluteus maximus (mW/kg)					Biceps femoris long head (mW/kg)				
	BW	UW	DW	FW	SW	BW	UW	DW	FW	SW
NE	72.9 ± 45.8	157.8 ± 101.3	41.8 ± 21.9	89 ± 54.3	63.4 ± 38.2	71.5 ± 33	116.1 ± 39.4	51.5 ± 20.6	91.8 ± 39.6	60.3 ± 20.4
ET	68.8 ± 43.7	159.5 ± 101.6	36.7 ± 20.7	82.6 ± 52.7	59.5 ± 36	76 ± 31.4	112 ± 34	61.8 ± 29.6	96.3 ± 38.4	61.3 ± 22
EA	73.7 ± 47.3	162.3 ± 106.2	40.7 ± 23.9	89.9 ± 55.9	62.9 ± 37.5	72 ± 30.8	110.2 ± 35.5	58.7 ± 29	101.2 ± 50.9	66.7 ± 27.3
AS	Rectus femoris (mW/kg)					Vastus medialis (mW/kg)				
	BW	UW	DW	FW	SW	BW	UW	DW	FW	SW
NE	198 ± 63.3	225.2 ± 69.7	230.1 ± 66.2	233.1 ± 72.1	188 ± 52.2	52.6 ± 21	84 ± 20.2	63.2 ± 27.2	66.6 ± 22.7	41.6 ± 11.2
ET	229.3 ± 70.9	244.7 ± 89.5	235.9 ± 78.9	238.9 ± 64.1	211.9 ± 75.1	60.8 ± 20.3	114.1 ± 22.1	72.1 ± 23	75.3 ± 28.8	49 ± 13.5
EA	205.5 ± 69.3	233.3 ± 91.5	236.5 ± 84.2	234.2 ± 87.1	207.3 ± 72.2	59 ± 13.7	108.7 ± 21.9	75.8 ± 22.9	78.7 ± 28.6	53.6 ± 22.1
AS	Medial gastrocnemius (mW/kg)					Soleus (mW/kg)				
	BW	UW	DW	FW	SW	BW	UW	DW	FW	SW
NE	197.1 ± 34.2	205.5 ± 36.1	155.3 ± 26.8	204.4 ± 39.9	160.9 ± 26.9	290.8 ± 67.4	355.4 ± 100.9	238.1 ± 63	334.8 ± 85.6	219.5 ± 73.8
ET	201.1 ± 44.9	198.2 ± 25	175.6 ± 37.3	221.2 ± 47.6	172 ± 30.3	318.3 ± 90	368.4 ± 108.9	250.5 ± 56.7	369 ± 89	243.3 ± 55.5
EA	198.2 ± 36.5	208.3 ± 32.2	163.4 ± 33	221.2 ± 69.2	176 ± 31.6	291.8 ± 83.8	385.1 ± 101.4	240.9 ± 88.3	354.3 ± 143.9	246.7 ± 65.2
